# A rare congenital malformation: caudal regression syndrome

**DOI:** 10.11604/pamj.2013.14.30.2364

**Published:** 2013-01-21

**Authors:** Cherkaoui Mandour, Brahim El Mostarchid

**Affiliations:** 1 Departement Of Neurosurgery, Military Hospital Mohammed V, Rabat, Morocco

**Keywords:** Caudal regression syndrome, congenital malformation, syringomyelia, vertebral agenesis

## Image in medicine

Caudal regression is a rare syndrome which has a spectrum of congenital malformations ranging from simple anal atresia to absence of sacral, lumbar and possibly lower thoracic vertebrae. It results from a disturbance in the fetal mesoderm in early pregnancy (< 4^th^ week of gestation). Maternal diabetes, genetic predisposition and vascular hypoperfusion have been suggested as possible causative factors but no true causative factor has been determined. Associated organ system dysfunction depends on the severity of the disease. Prenatal ultrasonographic diagnosis of this syndrome is possible at 22 weeks of gestation. We report a case of a four months old male newborn to a known diabetic mother.

**Figure 1:**
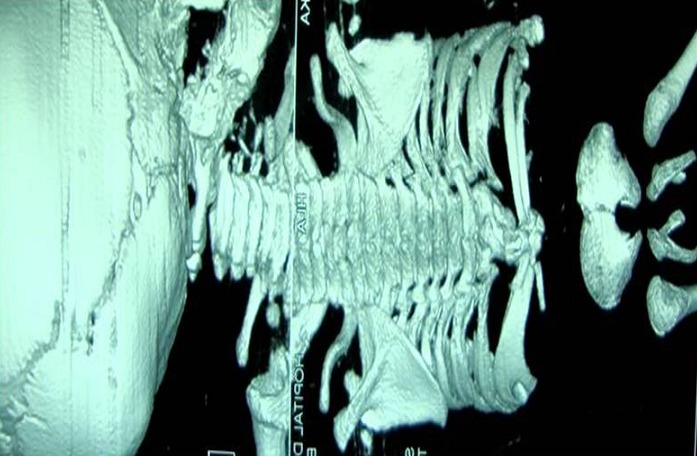
Bone reconstruction in 3D that shows a syringomyelia from D4 to D7; terminal myelocystocele (D10); agenesis of D11, D12, lumbar vertebrae, sacrum and coccyx; contiguous appearance of kidneys without prevertebral parenchymal bridge

